# Molecular detection of mixed infections with multiple dengue virus serotypes in suspected dengue samples in Tamaulipas, Mexico

**DOI:** 10.1590/0074-02760160468

**Published:** 2017-07

**Authors:** Rocío Requena-Castro, Miguel Ángel Reyes-López, Rosa Eminé Rodríguez-Reyna, Prisco Palma-Nicolás, Virgilio Bocanegra-García

**Affiliations:** Centro de Biotecnología Genómica, Instituto Politécnico Nacional, Reynosa, Tamaulipas, México

**Keywords:** serotypes, dengue, Flavivirus

## Abstract

This study aimed to detect dengue virus (DENV) serotypes in serum samples obtained in Matamoros Tamaulipas, Mexico, and to determine the concordance of conventional nested reverse transcriptase polymerase chain reaction (RT-PCR) and a serological test [enzyme-linked immunosorbent assay (ELISA NS1)]. Here, we detected mixed infections consisting of four serotypes of DENV. The most prevalent serotype was DENV-1, followed by DENV-4. This is the first report of DENV-4 in our region. Mixed infections were also detected in 21.5% of samples, and the predominant coinfection consisted of DENV-1 and DENV-2. Therefore, continuous epidemiological surveillance of DENV in this area is required to predict future forms of dengue heterologous infections and the effect of this on health care.

Dengue fever is a viral infection caused by the dengue virus (DENV), which belongs to the *Flaviviridae* family of the *Flavivirus* genus. It consists of four serotypes (DENV-1, -2, -3 and -4). These serotypes are closely related, but they display antigenic differences. Identifying DENV serotypes is very important because heterologous infections with Dengue Haemorrhagic Fever (DHF) are of great concern (Hammon et al. 1960). We examined a total of 243 sera samples from patients diagnosed with DHF according to the WHO SEARO (2011) criteria. These samples were obtained between August and October 2013 (106 men, 137 women) in Matamoros City, Mexico. For the enzyme-linked immunosorbent assay (ELISA), the commercial kit Panbio Dengue Early used for the detection of NS1 was employed. Reverse transcription was performed according to the manufacturer’s instructions (Kit ImProm-II Reverse Transcription System, Promega). Using polymerase chain reaction (PCR), a region of *CprM* DENV was amplified, and nested PCR was used to determine the specific serotype published by Lanciotti et al. (1992). Out of the 243 suspected dengue samples, 136 (56%) were positive by ELISA NS1, including 76 women and 60 men. The rest of the 107 (44%) samples were negative for the virus. Using PCR, only 71/243 (29.21%) were found to be positive, including 34 (47.8%) women and 37 (52.12%) men. No statistically significant association (p > 0.05) was found between dengue infections and gender. Samples obtained from people 16-30 years old showed the highest rate of dengue infection. Although the WHO SEARO (2011) indicates that the age groups most affected by dengue are infants, children and adults, our results agreed with Torres-Galicia et al. (2014), which found that the incidence of dengue and DHF has increased among young people. This indicates that dengue epidemiology is in flux, so epidemiological monitoring is still very important. Of the 136 samples identified as positive for dengue by ELISA, only 62 (45.5%) were confirmed using PCR. Meanwhile, of the 107 samples classified as negative for dengue by ELISA, only nine (8.41%) were positive by PCR (Kappa value 0.342). The WHO suggests that a single diagnostic test for dengue is not enough, so it is necessary to complement one test with another test (serological, molecular, viral isolation or sequencing) to confirm the infection status of a suspected sample. Our study supports the findings published by Castro-Jorge et al. (2010) in Brazil. This group argued that the best detection method combined molecular and serological tests. They found that if the day of disease onset is known, the NS1 protein may circulate in the blood for up to nine days after the onset of symptoms. However, *DENV* RNA only remains in the blood for one to five days. In some cases, the patient does not remember when the period of viraemia began, and it is usually difficult to obtain this data.

When performing PCR on dengue-negative samples by ELISA only nine were found to be positive (9/107; 8.41%). Therefore, 71/243 samples were positive by PCR, indicating that a third of the total samples were positive for DENV. This agreed with previous studies, including Saxena et al. (2008) and Khan et al. (2013), which found that even when a large number of samples were assayed, only one-third were positive. This indicated that the sensitivity of our diagnostic tests is in accordance with previous reports. The results of the detection assays for dengue serotypes is shown in Table. With respect to detecting dengue serotypes by PCR, we found four serotypes of dengue circulating in the population. Additionally, mixed infections consisting of patients harbouring more than two serotypes concurrently were observed. Mixed infections found in this study were: infections with two serotypes 17% (12/71), infections with three serotypes 3% (2/71) and one infection with four serotypes 1.5% (1/71) (Figure). Additionally, 6.5% (5/71) of the samples did not display infection with any specific serotype by PCR. This lack of amplification may be due to nonspecific amplification of DNA from another *Flavivirus* virus, such as East St. Louis and West Nile virus, which was mentioned by Ferraz et al. (2013). In Tamaulipas, there have been reported cases of West Nile virus (Fernández-Salas et al. 2007). Due to the extreme cross-reactivity of *Flavivirus* NS1 antigen in serological tests, several known *Flavivirus* strains in the area must be monitored*.* In Mexico, the incidence of dengue cases has decreased. However, since dengue a re-emerging disease, surveillance must be performed continually. This is especially important because all four serotypes were detected in this study. Furthermore, DHF has been reported in some states in Mexico, and serotypes DENV-1 and DENV-2 were recently reported in Tamaulipas in 2013 (data from Sistema Nacional de Vigilancia Epidemiológica. Available from: epidemiologia.salud.gob.mx/dgae/panodengue/intd_dengue.html). The identification of circulating serotypes of dengue is critical for minimising the risk that severe forms of dengue may spread in the population. This is because the virulence of the infecting serotype may vary. For instance, the Asian genotype DENV-2 is associated with haemorrhagic dengue. Additionally, the genotype known as ‘Sri-Lanka’ or DENV-3 is considered the most virulent of this serotype. Infection with DENV-1 followed by a subsequent co-infection with DENV-2 is associated with DHF. In Mexico, Torres-Galicia et al. (2014) reported that consecutive infections with DENV-1 / DENV-2, DENV-3 / DENV-2 or DENV-4 / DENV-2 serotypes are also associated with DHF. Another study in Peru reported mixed infections of the following serotype combinations: DENV-1 / DENV-2, DENV-1 / DENV 3, DENV-1 / DENV-4, DENV-2 / DENV-3, DENV-2 / DENV-4, DENV-3 / DENV -4, DENV-1 / DENV-2 / DENV-3 and DENV-1 / DENV-3 / DENV-4 (Mamani et al. 2010). Recently, Reddy et al. (2017) reported multiple dengue infection in 26 samples obtained in India. Although the DENV-1 and DENV-3 serotype co-infection was predominant, they also found co-infections with two or three other serotype combinations, in addition to two samples infected with DENV-1, 2, 3 and 4. This study mentioned that concurrent infections with two or more serotypes is an alarming new trend for dengue epidemiology in India. Therefore, detailed information regarding circulating serotypes can help identify geographical areas where DHF cases are likely to arise. We found that DENV-1 was the predominant serotype (29/71; 40.8%) and serotype DENV-4 (11/71; 15.5%) had not been previously reported in this area. Finally, this is the first study to report both mixed infections and the presence of these serotypes in Matamoros Tamaulipas, Mexico. Dengue fever remains a major global health concern, and the presence of mixed infections is concerning because severe forms of dengue can manifest from these coinfections. Early detection and proper monitoring of patients are necessary to identify the circulating dengue virus serotypes, aiding subsequent epidemiological studies and disease control measures.

**TABLE:** Distribution of dengue virus (DENV) serotypes

Infecting serotype detected by nested PCR		ELISA assays Samples		Gender		
	
		^a^P	^b^N	Men	Women	Total
Mono infection	Undetected	1	4	3	2	5
	Serotype 1	26	3	14	15	29
	Serotype 2	9	0	5	4	9
	Serotype 3	2	0	1	1	2
	Serotype 4	10	1	6	5	11
Double infection	Serotypes 1 and 2	9	0	4	5	9
	Serotypes 1 and 4	0	1	0	1	1
	Serotypes 2 and 4	2	0	2	0	2
Triple infection	Serotypes 1, 2 and 3	1	0	1	0	1
	Serotypes 1, 3 and 4	1	0	0	1	1
Quadruple infection	Serotypes 1, 2, 3 and 4	1	0	0	1	1

Total		62	9	36	35	71

^a^P: positive sample; ^b^N: negative sample; ELISA: enzime-linked immunosorbent assay; PCR: polymerase chain reaction.

**Figure:**
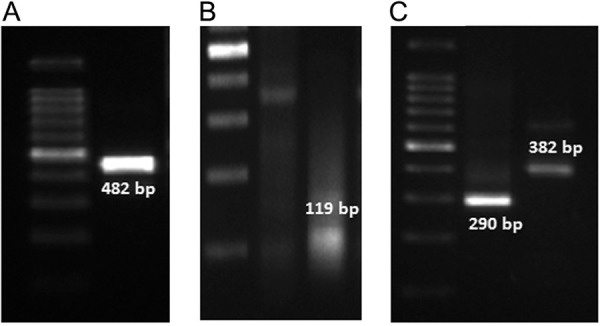
Agarose gel electrophoresis showing amplification of four dengue virus (DENV) serotypes in one co-infected patient sample (ID Patient D32). (A) Lane 1: 100-bp marker; Lane 2: amplification of *DENV-1* (482 bp). (B) Lane 1: 100-bp marker; Lane 2: sample negative for *DENV-2*; Lane 3: sample positive for *DENV-2* (119 bp). (C) Lane 1: 100-bp marker; Lane 2: amplification of *DENV-3* (290 bp); Lane 3: amplification of *DENV-4* (389 bp).
